# C/EBPα-mediated ACSL4-dependent ferroptosis exacerbates tubular injury in diabetic kidney disease

**DOI:** 10.1038/s41420-024-02179-w

**Published:** 2024-10-23

**Authors:** Ziru Xia, Zhaonan Wei, Xin Li, Yunzi Liu, Xiangchen Gu, Jianhua Tong, Siyi Huang, Xiaoyue Zhang, Weiming Wang

**Affiliations:** 1 Department of Nephrology, Institute of Nephrology, Ruijin Hospital, Shanghai Jiao Tong University School of Medicine, Shanghai, 200025 People’s Republic of China; 2 Institute of Nephrology, Shanghai Jiao Tong University, School of Medicine, Shanghai, China; 3 Department of General Internal Medicine, West China Second University Hospital, Sichuan University, Chengdu, 610041 China; 4 Department of Nephrology, Yueyang Hospital of Integrated Traditional Chinese and Western Medicine, Shanghai University of Traditional Chinese Medicine, Shanghai, 200437 People’s Republic of China; 5 Faculty of Medical Laboratory Science, Central Laboratory, Ruijin Hospital, Shanghai Jiao Tong University School of Medicine, Shanghai, 200025 People’s Republic of China

**Keywords:** Chronic kidney disease, Cell death

## Abstract

Diabetic kidney disease (DKD) is a prevalent and debilitating complication of diabetes characterized by progressive renal function decline and a lack of effective treatment options. Here, we investigated the role of the transcription factor CCAAT/enhancer binding protein alpha (C/EBPα) in DKD pathogenesis. Analysis of renal biopsy samples revealed increased C/EBPα expression in patients with DKD. Using RNA sequencing and proteomics, we explored the mechanisms through which the C/EBPα contributes to DKD. Our findings demonstrated that C/EBPα exacerbated tubular injury by promoting acyl-CoA synthetase long-chain family member 4 (ACSL4)-dependent ferroptosis. We identified that C/EBPα upregulated ACSL4 expression by binding to its transcription regulatory sequence (TRS), leading to elevated lipid peroxidation and ferroptosis. Furthermore, inhibition or genetic ablation of C/EBPα attenuated ferroptosis and mitigated tubular injury in DKD. These results highlighted the C/EBPα-ACSL4-ferroptosis pathway as a promising therapeutic target for DKD treatment.

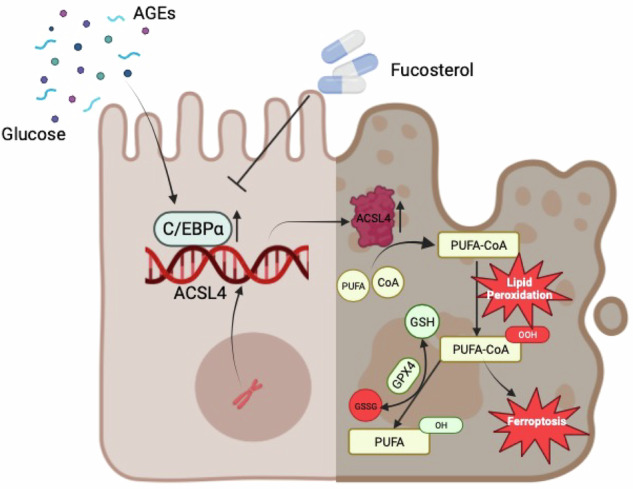

## Introduction

Diabetic kidney disease (DKD) is a kidney disorder that arises as a complication of diabetes, imposing a significant burden on healthcare [1]. It is characterized by damage to kidney blood vessels, resulting in protein leakage, hypertension, and reduced renal function [2]. While metabolic disorders and inflammatory infiltration have been implicated in DKD progression, the primary factors and underlying mechanisms driving its advancement remain under investigation [3].

Recent studies have highlighted the role of transcription factors such as CCAAT/enhancer binding protein alpha (C/EBPα) in DKD progression [4]. C/EBPα, a member of the CEBP family, contains a basic region leucine zipper (bZIP) motif and generates two isoforms, p42 and p30, based on translational initiation sites within the CEBPA mRNA [5]. The p30 isoform primarily inhibits terminal cell differentiation, whereas the p42 isoform regulates cell proliferation and differentiation [6, 7]. C/EBPα is known for its involvement in myeloid cell differentiation [8, 9], acute myeloid leukemia (AML) [6, 10] and various metabolic processes [11, 12], although the detailed mechanisms remain incompletely understood.

In this study, we investigated the upregulation of C/EBPα in renal tissues from DKD patients and in a mouse model of DKD induced by streptozotocin (STZ). Specific knockout of C/EBPα in tubular cells significantly mitigated renal fibrosis and inflammation, halting DKD progression. Using comprehensive RNA sequencing and proteomic analysis, we identified ferroptosis as a pivotal pathway, with acyl-CoA synthetase long-chain family member 4 (ACSL4) playing a crucial role in driving ferroptosis associated with C/EBPα.

Ferroptosis is an iron-dependent cell death process characterized by lipid peroxide accumulation [13]. Studies underscored the importance of lipid metabolism homeostasis in preserving renal function in DKD patients [14], where ACSL4-mediated lipid peroxidation and reactive oxygen species (ROS) release are critical factors [15–17].

Furthermore, our study revealed that overexpression of Cebpa exacerbated tubular injury and ferroptosis in DKD mice, whereas the administration of the C/EBPα inhibitor Fucosterol protected db/db mice from renal fibrosis and preserved renal function. These findings collectively suggest that targeting C/EBPα to modulate ACSL4-induced ferroptosis could be a promising therapeutic strategy for DKD.

## Results

### Elevated C/EBPα expression in diabetic kidney disease

**Fig. 1:**
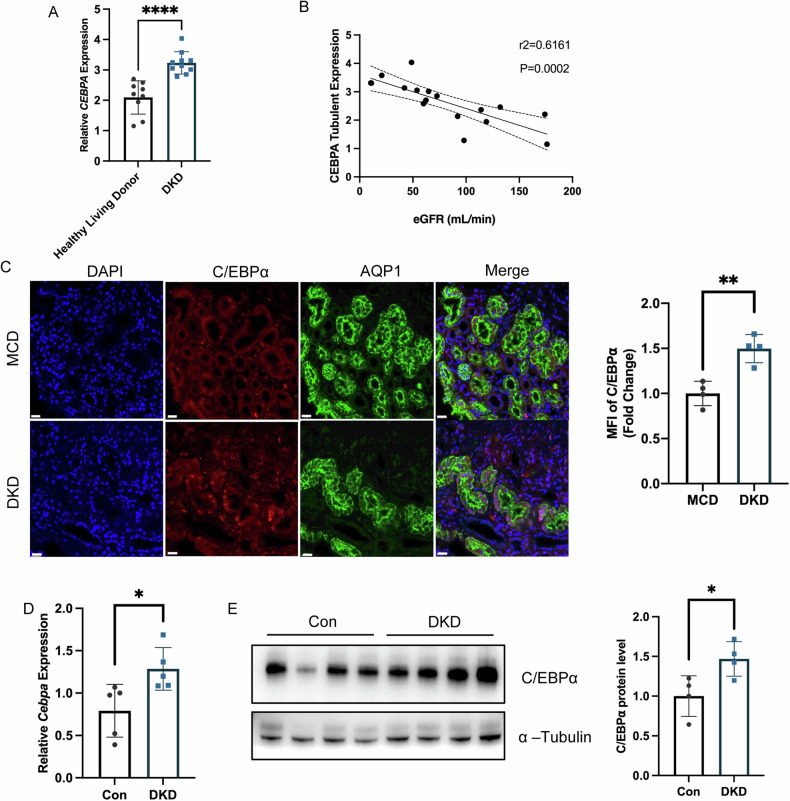
Increased C/EBPα expression in diabetic kidney disease. **A** Data form Nephroseq (https://www.nephroseq.org) revealed elevated *CEBPA* mRNA levels in the kidney cortex of DKD patients compared to healthy living donors (*n* = 9–10 patients per group). **B** A correlation analysis between eGFR and *CEBPA* expression. (*n* = 17 patients). **C** Representative images of triple immunofluorescence staining of the proximal tubular marker aquaporin 1 (AQP1), C/EBPα, and DAPI (nuclei), as well as merged images of all three channels. Scale bar: 20 μm. The bar graph shows the quantification of the mean ± SD. (*n* = 4 patients per group; 10 independent fields per section were evaluated). **D** RT-qPCR analysis showed increased *Cebpa* mRNA levels in the kidney cortex in DKD mice compared to control mice. (*n* = 5 mice per group). **E** Immunoblot and statistical analysis showing increased C/EBPα protein levels in the kidney cortex of DKD mice. (*n* = 4 mice per group). The data are presented as the mean ± SD; *P < 0.05, **P < 0.01 compared with the control.

In our study, we investigated the expression of CEBPA in patients with Diabetic Kidney Disease (DKD) using the Neproseq dataset. We observed a significant upregulation of *CEBPA* mRNA levels in the kidneys of DKD patients compared to those of healthy living donors (Fig. 1A). Additionally, we identified a noteworthy correlation between estimated glomerular filtration rate (eGFR) and *CEBPA* expression (Fig. 1B). To further characterize the role of C/EBPα, we conducted a comparative analysis of its expression in DKD and Minimal Change Disease (MCD). The results revealed more intense C/EBPα staining in the proximal tubular region of DKD kidneys (Fig. 1C). Subsequently, we investigated C/EBPα expression in the renal cortex of a DKD mouse model, confirming its presence at both the mRNA (Fig. 1D) and protein levels (Fig. 1E). Similar upregulation was observed in mouse primary tubular epithelial cells (PTECs) exposed to high glucose (Fig. S2A, B). PTECs also showing a dose-dependent relationship between C/EBPα expression and Advanced Glycation End-products (AGEs) concentration (Fig. S2C).These findings collectively indicated a strong correlation between increased C/EBPα expression and the development of DKD.

### Specific tubular deletion of C/EBPα alleviated renal injury

To investigate the role of tubular C/EBPα in diabetic nephropathy, we generated Pepck-Cre *Cebpa*^fl/fl^ mice (Cre^+^/*Cebpa*^fl/fl^ mice). Under control conditions, Cebpa deletion did not significantly alter body weight (Fig. S3A), blood glucose levels (Fig. S3B), or kidney weight-to-body weight ratio (Fig. S3C) in mice.

**Fig. 2:**
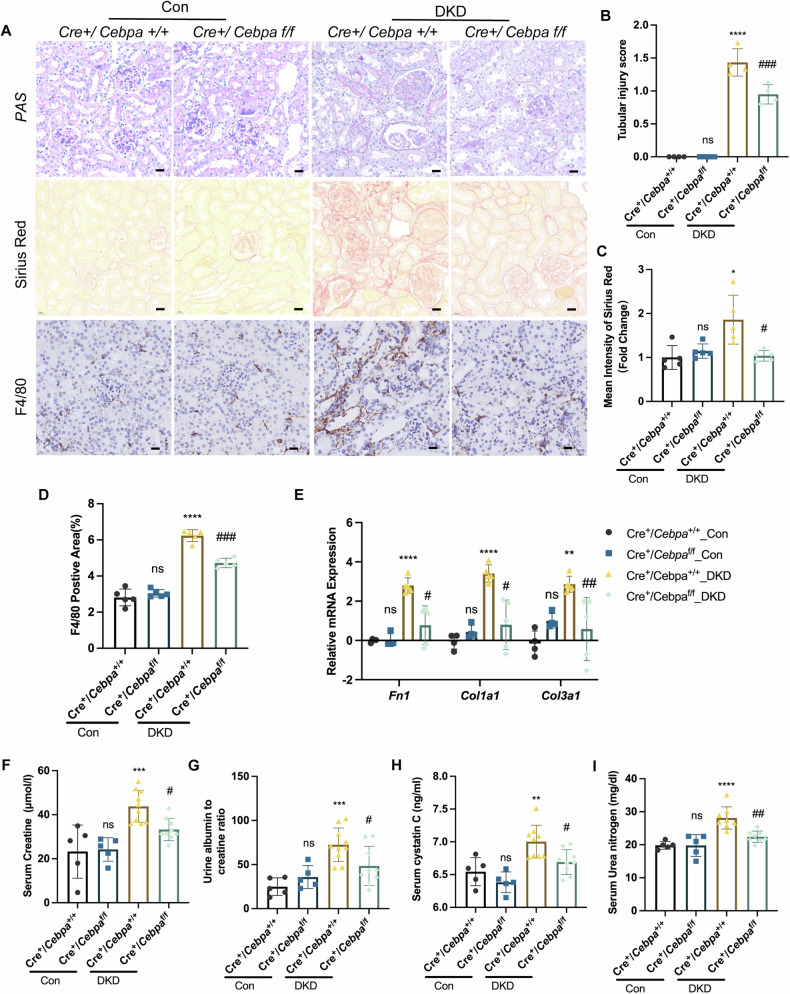
Specific tubular deletion of C/EBPα alleviated renal injury. **A** Representative microphotographs of PAS staining, Sirius red staining and F4/80 IHC staining of kidney sections. Scale bar: 20 μm. **B** The bar graph shows the tubular injury score. (*n* = 4 mice per group; 10 independent fields per section were examined). **C** The bar graph shows the mean intensity of Sirius red staining. (*n* = 5 mice per group; 10 independent fields per section were examined). **D** The bar plot shows the quantification of the F4/80-positive area. (*n* = 5 mice per group; 10 independent fields per section were examined). **E** mRNA levels of renal fibrosis-associated genes determined by RT‒qPCR in mice. (*n* = 4–5 mice per group). **F–I** Serum creatinine concentrations (**F**), urine albumin-to-creatinine ratios (**G**), serum cystatin C concentrations (**H**), and serum urea nitrogen concentrations (**I**) in WT and *Cebpa*-KO mice with DKD (*n* = 6–11 mice per group). The data represent the means ± SDs. ns: no significant difference, **P < 0.01, ***P < 0.001, ****P < 0.0001 compared with the respective experimental control conditions; ^#^*P* < 0.05, ^##^*P* < 0.01, ^####^*P* < 0.0001 compared with the WT mice under the same experimental conditions.

Under DKD conditions, Cre^+^/Cebpa^+/+^ (WT) mice exhibited more severe pathological changes, including marked flattening, vacuolation, and loss of the brush border in tubular epithelial cells, leading to an increased tubular injury score (Fig. 2A and B). Sirius Red staining showed increased renal fibrosis (Fig. 2A and C), and F4/80 immunohistochemical staining revealed significant macrophage infiltration in WT mice compared with KO mice (Fig. 2A and D). Gene expression analysis revealed elevated mRNA levels of the renal fibrotic markers, including *Fn1, Col1a1* and *Col3a1* (Fig. 2E), and tubular injury marker *Havcr1* (Fig. S3D) in DKD WT mice compared to DKD Cre + /Cebpa^fl/fl^ (KO) mice. Moreover, WT mice exhibited significant deterioration of renal function, as indicated by elevated serum creatinine (SCr) levels (Fig. 2F), an increase in the urinary albumin-to-creatinine ratio (UACR) (Fig. 2G), elevated serum cystatin C levels (Fig. 2H), and increased serum urea nitrogen levels (Fig. 2I). Overall, our findings demonstrated that specific tubular deletion of C/EBPα alleviated renal injury and inflammation in mice with DKD, underscoring its critical role in the pathogenesis of diabetic nephropathy.

### Specific tubular deletion of C/EBPα mitigated ferroptosis in DKD mice

**Fig. 3:**
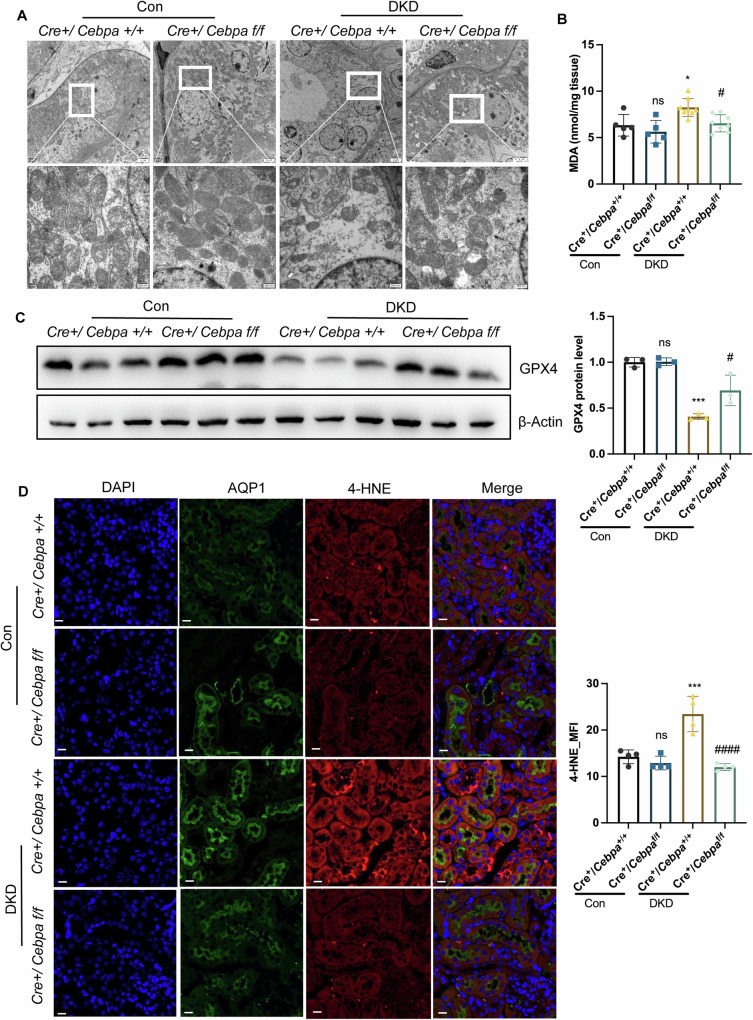
C/EBPα induced tubular injury through ferroptosis. **A** Representative electron microscopy images of cortical proximal tubules from control and C/EBPα f/f mice subjected to DKD. Scale bars: 2 μm (upper panels) and 500 nm (lower panels). **B** MDA levels (malondialdehyde) in DKD WT and *Cebpa*-KO mice. (*n* = 5–8 mice per group) (**C**) Immunoblot and statistical analysis of GPX4 protein levels in the kidney cortex of control and DKD WT and *Cebpa*-KO mice. (*n* = 3 per group). **D** Representative images of triple immunofluorescence staining of the proximal tubular marker aquaporin 1 (AQP1), 4-Hydroxynonenal, and DAPI (nuclei); and merged images for all three channels. Scale bar: 20 μm. The bar graph shows the quantification of the mean intensity of 4-HNE. (*n* = 4 mice per group; 10 independent fields per section were analyzed.) The bar graphs represent the means ± SDs. ns: no significant difference, *P < 0.05, **P < 0.01, ***P < 0.001, ****P < 0.0001 compared with their corresponding control kidneys; ^#^*P* < 0.05, ^##^*P* < 0.01.

Further analysis involving the C/EBPα inducer ICCB280 and RNA sequencing suggested the potential involvement of C/EBPα in the ferroptosis pathway (Fig. S4A). Supporting this discovery, observations of DKD WT mice revealed mitochondrial crumpling with disrupted ridges (Fig. 3A) and increased levels of malondialdehyde (MDA) (Fig. 3B). Moreover, there was a notable decrease in the expression of the enzyme GPX4, crucial for converting GSSG to GSH, in DKD WT mice compared to their KO counterparts (Fig. 3C). Importantly, there were no significant differences in GPX4 mRNA levels among the groups (Fig. S3E), indicating a functional decrease rather than transcriptional modulation. Immunofluorescence staining of 4-hydroxynonenal (4-HNE), a lipid peroxidation product, revealed significantly increased fluorescence intensity in the kidneys of DKD WTmice (Fig. 3D). These findings confirmed the critical role of C/EBPα in ferroptosis.

### C/EBPα deficiency alleviated ferroptosis in DKD by downregulating ACSL4

**Fig. 4:**
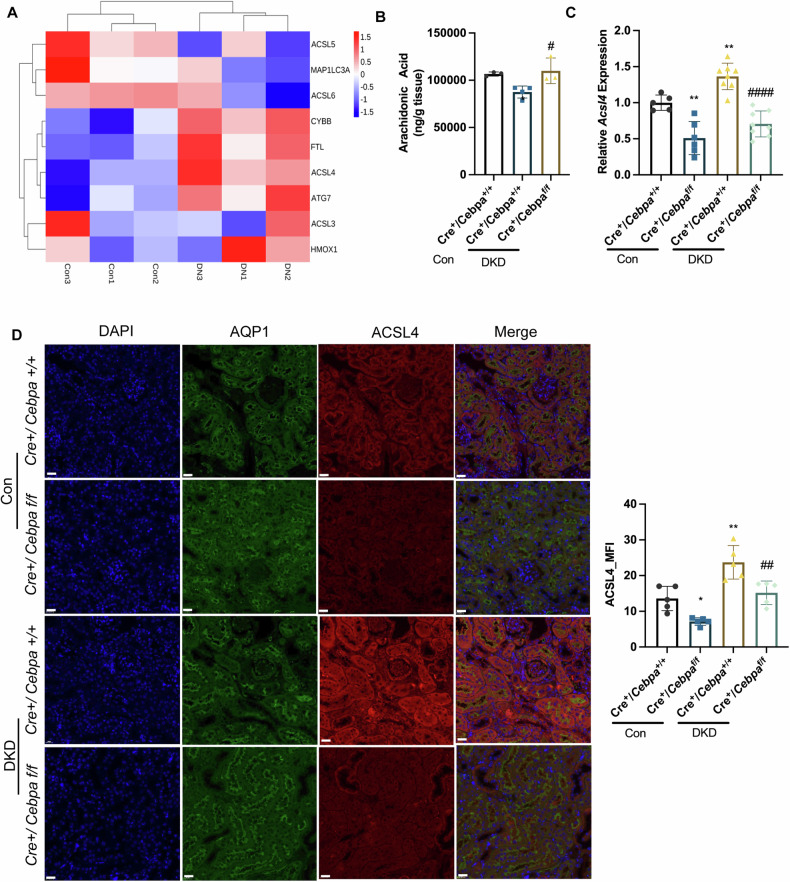
C/EBPα deficiency alleviated ferroptosis in DKD by downregulating ACSL4. **A** Heatmap showing ferroptosis-related genes that were differentially expressed in DKD mice and were regulated by C/EBPα, as determined by RNA sequencing. **B** Arachidonic acid levels in the kidney tissues of DKD WT and *Cebpa*-KO mice. (*n* = 3-4 mice per group). **C** RT‒qPCR analysis of *Acsl4* mRNA levels in kidneys from WT and *Cebpa*-KO mice. (*n* = 5–8 per group). **D** Representative images of triple immunofluorescence staining of the proximal tubular marker aquaporin 1 (AQP1), ACSL4, and DAPI (nuclei) and merged images of all three channels. Scale bar: 20 μm. The bar graph shows the quantification of the mean intensity of ACSL4. (*n* = 5 mice per group; 10 independent fields per section were examined). Bar graphs represent the mean ± SD; *P < 0.05, **P < 0.01, and ***P < 0.001 compared with the respective experimental controls; ^#^*P* < 0.05 compared with cells or mice under the same experimental conditions.

To investigate the key molecules involved in the impact of C/EBPα on ferroptosis, we performed a tandem mass tag (TMT) proteomic analysis of renal tissues from mice with diabetic kidney disease (DKD) and untreated Cre^+^/Cebpa^+/+^ (wild-type) mice. We focused on proteins related to the ferroptosis pathway and identified those elevated in DKD. The resulting heatmap revealed a significant increase in the protein levels of ACSL4 (Fig. 4A), a pivotal enzyme regulated by C/EBPα involved in PUFA-CoA formation, in the kidney tissue of DKD mice. Metabolomic analysis of medium-to-long-chain fatty acids (Fig. 4B) demonstrated a decrease in arachidonic acid levels, a substrate of ACSL4, in the kidneys of DKD WT mice, while arachidonic acid was increased in DKD KO mice, suggesting opposing effects on ACSL4 expression in the kidney. These findings were validated by Western blot analysis, which showed markedly lower ACSL4 protein levels (Fig. S4B) in the kidneys of KO mice compared to WT mice. Additionally, qPCR (Fig. 4C) and immunofluorescence staining (Fig. 4D), confirmed significant increases in the ACSL4 mRNA expression and mean fluorescence intensity in the kidneys of DKD WT mice, whereas levels remained stable and lower in DKD KO mice.

### C/EBPα overexpression exacerbated ferroptosis in DKD mice via ACSL4 upregulation

**Fig. 5:**
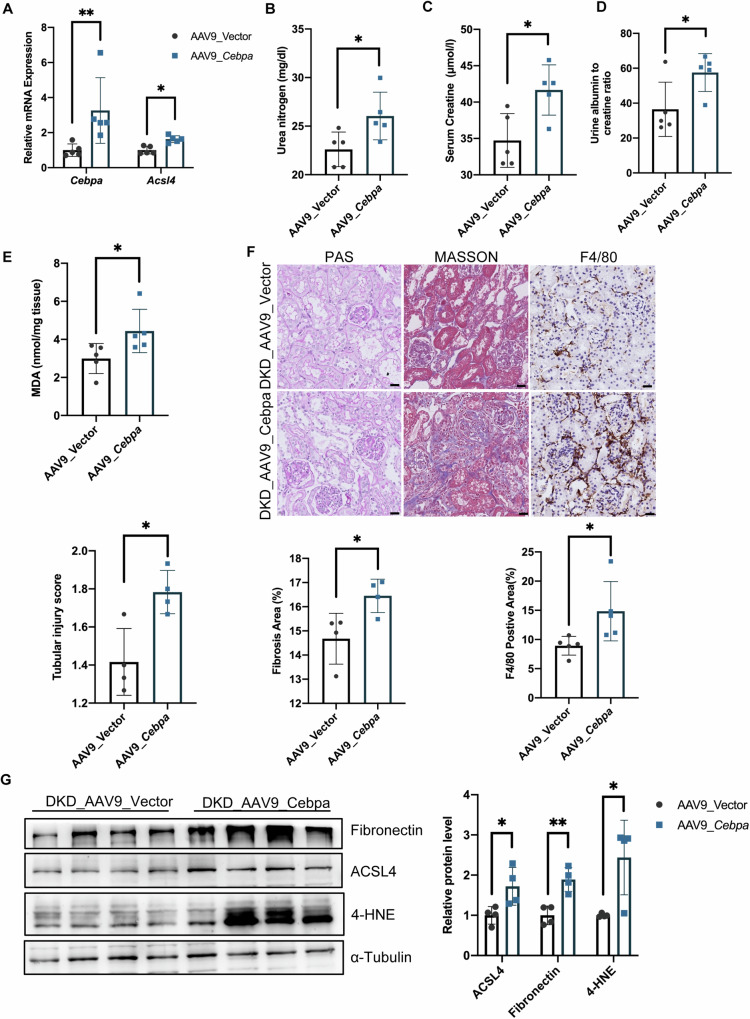
C/EBPα overexpression exacerbated ferroptosis in DKD mice via ACSL4 upregulation. **A** The mRNA levels of *Cebpa* and *Acsl4* in the kidneys of DKD mice injected with the AAV9 vector (control) or the AAV9-*Cebpa* plasmid (*Cebpa* overexpression). **B**–**E** Blood urea nitrogen (**B**), serum creatinine (**C**), urine albumin-to-creatinine ratio (UACR) (**D**), and Malondialdehyde (MDA) (**E**) levels in DKD control and *Cebpa*-overexpressing mice. (*n* = 5 mice per group) (**F**) Representative micrographs showing PAS staining, Masson’s trichrome staining, and F4/80 expression in kidney sections from mice treated as described above. Scale bar: 20 μm. The bar graph shows the quantification of the tubular injury score, fibrosis area and percentage of F4/80-positive stained area. (*n* = 4–5 mice per group; 10 independent fields per section were examined). **G** Immunoblot and statistical analysis of fibronectin, ACSL4 and 4-HNE levels in the kidney cortex of DKD mice injected with the AAV9 vector or AAV9-*Cebpa* plasmid. (*n* = 4 per group). ns: no significant difference, *P < 0.05, **P < 0.01, ***P < 0.001 compared with the respective experimental controls; ^#^*P* < 0.05, ^###^*P* < 0.0001 compared with cells under the same experimental conditions.

To investigate the impact of C/EBPα overexpression in the kidney cortex on diabetic nephropathy, we established a Cebpa-overexpressing animal model using an AAV9 vector (Fig. S4C) and evaluated its effects on kidney function in DKD mice. Our findings revealed that AAV9-Cebpa vector administration led to increased levels of *Cebpa* and *Acsl4* mRNA (Fig. 5A), accompanied by elevated levels of urea nitrogen (Fig. 5B), serum creatinine (Fig. 5C), UACR (Fig. 5D), and MDA concentration (Fig. 5E) in DKD mice. Additionally, we observed more serious renal injury, fibrosis, and inflammatory cell infiltration in the AAV9-Cebpa group (Fig. 5F). Furthermore, there were significant increases in mRNA expression of *Lcn2* (Fig. S4D), fibrosis-related factors (Fig. S4E), and notable elevation in ACSL4, Fibronectin and 4-HNE levels in the kidneys of Cebpa-overexpressing mice (Fig. 5G). These findings indicated that upregulating C/EBPα in the kidney cortex modulated ACSL4 expression and exacerbated ferroptosis, thereby contributing to kidney damage in DKD mice.

### C/EBPα deficiency alleviated ferroptosis in PTECs by downregulating ACSL4

**Fig. 6:**
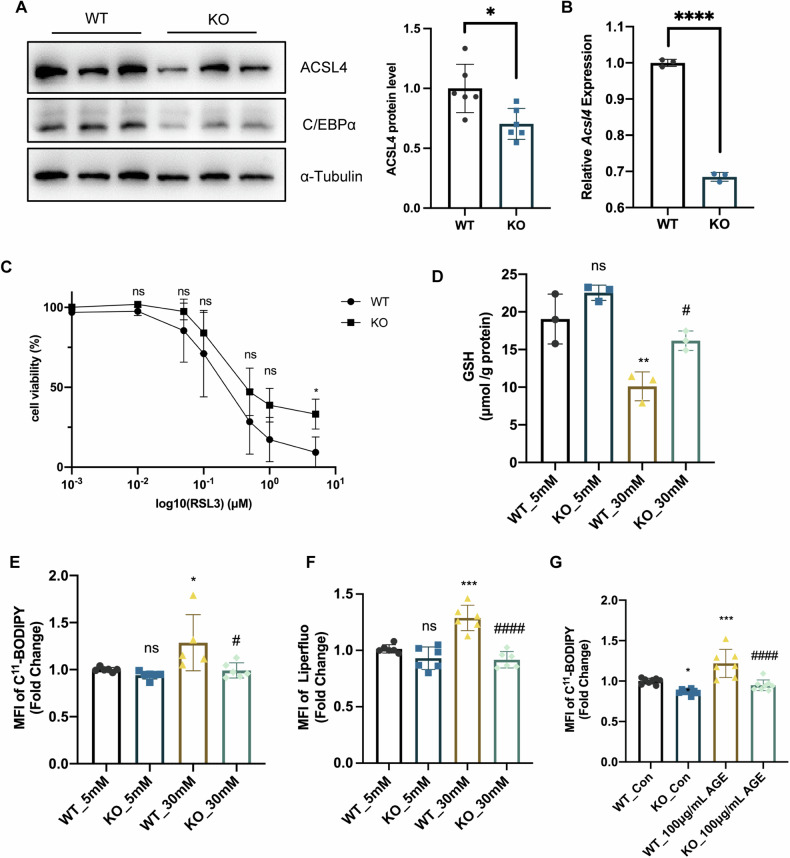
C/EBPα deficiency alleviated ferroptosis in PTECs by downregulating ACSL4. **A** Immunoblot and bar plot showing C/EBPα and ACSL4 protein levels in WT and KO PTECs. (*n* = 6 per group). **B** RT‒qPCR analysis of *Acsl4* mRNA levels in WT and KO PTECs. (*n* = 3 per group). **C** Viability of WT and KO PTECs treated with RSL-3 for 24 h. (*n* = 5 per group). **D** GSH levels in WT and *Cebpa*-KO PTECs treated with 30 mM D-glucose. (*n* = 3 per group). **E**, **F** C^11^-BODIPY(**E**) and Liperfluo (**F**) fluorescence intensity in D-glucose-treated WT and *Cebpa*-KO PTECs. (*n* = 4-6 per group). **G** C^11^-BODIPY fluorescence intensity in AGEs-treated WT and *Cebpa*-KO PTECs. (*n* = 4–6 per group). Bar graphs represent the mean ± SD; *P < 0.05, **P < 0.01, and ***P < 0.001 compared with the respective experimental controls; ^#^*P* < 0.05 compared with cells or mice under the same experimental conditions.

We examined PTECs from WT and KO mice and observed significantly lower protein and mRNA levels of CEBPA and ACSL4 in KO PTECs (Fig. 6A, B). Furthermore, KO PTECs exhibited higher cell viability following RSL-3 stimulation (Fig. 6C). Under stimulation with 30 mM D-glucose, KO PTECs showed elevated levels of glutathione (GSH) (Fig. 6D) and lower fluorescence intensities of C^11^-BODIPY and Liperfluo as detected by flow cytometry (Fig. 6E, F). Additionally, compared to WT PTECs, KO PTECs displayed reduced C^11^-BODIPY fluorescence in response to AGE stimulation (Fig. 6G). These findings indicate that C/EBPα plays a critical role in regulating ACSL4 expression and ferroptosis in tubular epithelial cells, with C/EBPα knockout providing protection against oxidative stress-induced damage.

### C/EBPα induced ferroptosis in tubular epithelial cells by upregulating ACSL4

**Fig. 7:**
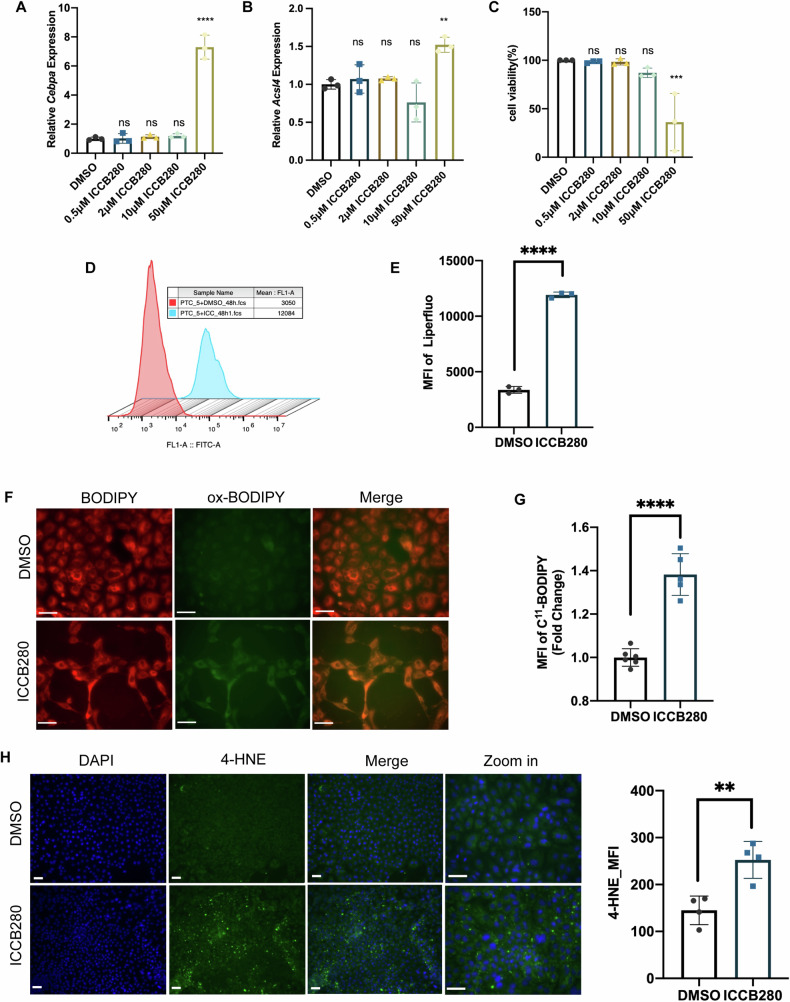
C/EBPα induced ferroptosis in tubular epithelial cells by upregulating ACSL4. **A**, **B** The mRNA level of *Cebpa* (**A**) and *Acsl4* (**B**) of PTECs treated with 0 to 50 μM ICCB280. (*n* = 3 per group). **(C)** viability of PTECs treated with 0 to 50 μM ICCB280. (*n* = 3 per group). (**D** and **E**) Representative and Bar graph showing the Liperfluo fluorescence intensity of PTECs treated with ICCB280. (*n* = 3 per group). **F**, **G** Representative C^11^-BODIPY staining (**F**) and bar graph showing the C^11^-BODIPY fluorescence intensity (**G**). (*n* = 5 per group) (**H**) Representative images of double immunofluorescence staining of PTECs for 4-hydroxynonenal and DAPI (nuclei); merge of the 2 channels and bar graph of 4-HNE fluorescence intensity. Scale bar: 30 μm. (*n* = 4 per group; 10 independent fields per section were examined). Bar graphs represent the mean ± SD; ns: no significant difference; ***P < 0.001 compared with the control; ****P < 0.0001 compared with the control.

To further validate our hypothesis, we used ICCB280 to induce C/EBPα expression in PTECs. qPCR analysis showed significant upregulation of *Cebpa* (Fig. 7A) and Acsl4 (Fig. 7B) mRNA expression in PTECs in response to 50 μM ICCB280 stimulation. Cell viability assays demonstrated dose-dependent reductions in viability in PTECs (Fig. 7C) upon ICCB280 treatment, which a 50% decrease observed at 50 μM ICCB280 compared to the control group. Flow cytometric analysis using Liperfluo (Fig. 7D, E), a specific probe for phospholipid peroxidation, and C^11^-BODIPY (Fig. 7F, G), a probe for lipid peroxidation, showed significant changes in the mean fluorescence intensity for both probes after ICCB280 stimulation. Additionally, ICCB280 treatment significantly decreased GSH concentration in PTECs (Fig. S5B), and robustly increased MitoSOX fluorescence intensity (Fig. S5C and S5D), indicating enhanced ferroptosis. These findings were further confirmed in PTECs, which exhibited higher 4-HNE production in response to C/EBPα induction compared to the DMSO control (Fig. 7H).

In PTECs transfection with the pcDNA3.1-*Cebpa*-3xFlag vector, we observed increased mRNA and protein levels of three key enzymes involved in PUFA peroxidation: *Acsl4, Por*, and *Lpcat3* (Fig. S6A, B). Furthermore, C/EBPα overexpression exacerbated lipid peroxidation in AGE- and high glucose-treated PTECs (Fig. S6C, D), and this change was accompanied by a decrease in GSH concentrations (Fig. S6E).

### C/EBPα regulated ACSL4 expression through direct binding to the TRS

**Fig. 8:**
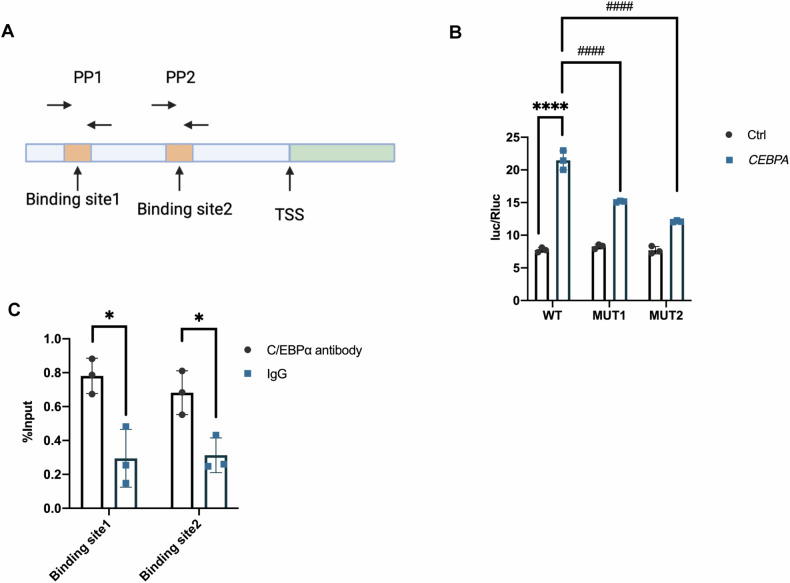
C/EBPα regulated ACSL4 expression by directly binding to the TRS of ACSL4. **A** Schematic diagram showing two possible binding sites (PP1 and PP2) of C/EBPα on the ACSL4 TRS. TSS: Translational start site. **B** Bar plot showing the Luc/Ruc ratio in HKK293 cells cotransfected with the different plasmids. NC: normal control; WT: wild-type vector with the whole ACSL4 TRS sequence; MUT1: vector with a mutation in binding site 1; MUT2: vector with a mutation in binding site 2. **C** ChIP‒qPCR analysis using an anti-C/EBPα antibody and the primer pairs shown in (**A**). The bar graphs represent the mean ± SD. *P < 0.05, ****P < 0.0001 compared with the respective experimental control; ^####^*P* < 0.0001 compared with cells under the same experimental conditions (*n* = 3 per group).

To investigate whether the transcription factor C/EBPα directly regulates ACSL4 expression, we performed luciferase assays and chromatin immunoprecipitation quantitative PCR (ChIP–qPCR) experiments. Using JASPR tools, we identified the two most likely binding sites of C/EBPα on the TRS sequence of ACSL4 (Fig. 8A). Plasmids containing mutant TRS sequences were constructed and separately transfected into HEK293 cells with wild-type or mutant plasmids and the pcDNA3.1-CEBPA-3xFlag vector. Dual-luciferase reporter analysis revealed that mutations in the binding sites significantly reduced the transcriptional activity of C/EBPα (Fig. 8B). Two pairs of primers for the binding sites were designed, and ChIP‒qPCR was performed to confirm our results (Fig. 8C). DNA at the binding sites was significantly increased in the C/EBPα antibody group compared to the IgG group, confirming that C/EBPα could directly bind to the promoter region of ACSL4 and regulate ACSL4 transcription.

### Fucosterol inhibited C/EBPα and relieved tubular injury in db/db mice

**Fig. 9:**
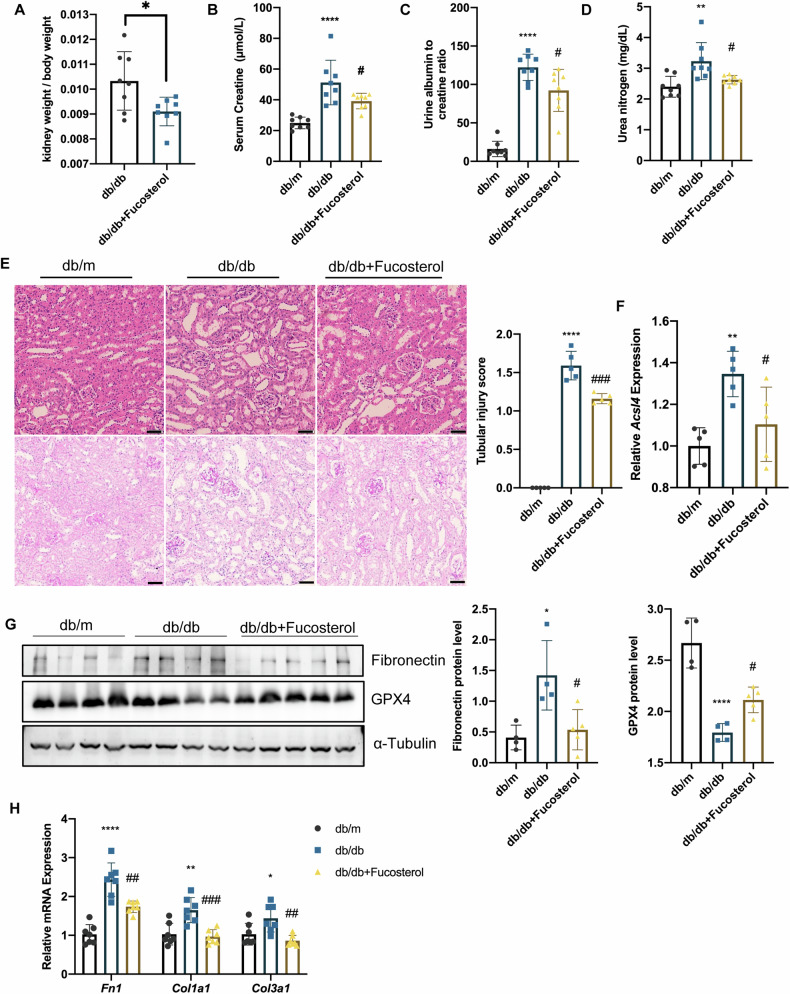
Fucosterol, which is a potential inhibitor of C/EBPα, relieves tubular injury in db/db mice. **A** Kidney weight/body weight ratios of db/db and db/db mice treated with 20 mg/kg Fucosterol for 6 weeks. **B**–**D** Serum creatinine levels (**B**), the urine albumin-to-creatinine ratio (**C**), and blood urea nitrogen levels (**D**) in the mice. **E** Representative micrographs showing H&E and PAS staining of kidney sections from mice treated with Fucosterol. Scale bar: 50 μm. The bar graph shows the tubular injury score. (*n* = 5 mice per group; 10 independent fields per section were examined). **F** The mRNA levels of *Acsl4* in the kidney tissues of the mice. (*n* = 5 mice per group) (**G**) Immunoblot and bar plot showing fibronectin and GPX4 protein levels in kidney tissues from db/db mice treated with Fucosterol. (*n* = 4–5 mice per group) (**H**) The mRNA levels of *Fn1, Col1a1* and *Col3a1* in the kidney tissues of the mice. (*n* = 7-8 mice per group) ns: no significant difference, *P < 0.05, **P < 0.01, ***P < 0.001, ****P < 0.0001 compared with the respective experimental control conditions; ^#^*P* < 0.05, ^##^*P* < 0.01, ^###^*P* < 0.001, ^####^*P* < 0.0001 compared with mice under the same experimental conditions.

In previous studies, Fucosterol, a natural extract of algae, has been demonstrated as a C/EBPα inhibitor in metabolic syndrome [18]. Based on these findings, our research aimed to examine the protective effects of Fucosterol against ferroptosis in the kidneys during diabetic nephropathy by inhibiting C/EBPα. To investigate this phenomenon, 8-week-old db/m and db/db mice were randomly assigned to three treatment groups and treated for 6 weeks: db/m, db/db, or db/db + Fucosterol. Our results indicated that Fucosterol treatment reduced the kidney/body weight ratio (Fig. 9A). These findings were further supported by reductions in serum creatinine levels (Fig. 9B), UACR (Fig. 9C), urea nitrogen levels (Fig. 9D), and tubular injury observed in PAS sections (Fig. 9E).

Additionally, db/db mice exhibited a notable increase in ACSL4 and more severe GPX4 consumption (Fig. 9F, G), indicating ferroptosis. Treatment with Fucosterol effectively alleviated ferroptosis in db/db mice. Furthermore, the protein level of Fibronectin (Fig. 9G) and the mRNA levels of *Fn1, Col1a1*, and *Col3a1* (Fig. 9H) were significantly higher in the db/db group than in the db/db + Fucosterol group.

## Discussion

DKD is a multifaceted condition influenced by various genes. Recent analysis of public datasets has demonstrated the strong correlation between the expression of transcription factors, including C/EBPα, and tubulointerstitial injury in diabetes [4]. Consistent with these findings, our study showed the upregulation of C/EBPα specifically within the proximal tubular cells of both DKD patients and mice. C/EBPα, a well-known transcription factor, plays significant roles in biological processes such as adipocyte differentiation and cell cycle regulation [19, 20]. However, its role in the kidney appears to be cell specific. For example, in podocytes, C/EBPα protects against inflammation and injury in certain nephropathies [21, 22]. Conversely, in renal endothelial cells, C/EBPα promotes TGFβ-induced endothelial-to-mesenchymal transition, leading to renal fibrosis [23]. Despite its prevalence in approximately 80% of renal cells, the role of C/EBPα in tubular epithelial cells has largely not been examined.

Ferroptosis, a distinct form of cell death first identified in cancer cells, has gained importance in renal diseases [24]. The key mechanisms involve the accumulation of lipid peroxides and disruptions in iron metabolism [25]. ACSL4, which is a critical enzyme in ferroptosis, catalyzes the transformation of PUFA and CoA to form PUFA-CoA [14, 16, 26, 27]. In acute renal injury (AKI), genetic deletion or inhibition of Acsl4 significantly reduces serum creatinine levels in mice [28, 29]. In chronic kidney disease (CKD), ferroptosis contributes to the progression of tubulointerstitial fibrosis, which is a common outcome [30, 31]. Recent studies have also highlighted the predictive value of ACSL4 expression in DKD [32]. However, there is a lack of fundamental research focusing on ACSL4 and its transcriptional regulation in the context of DKD.

Intriguingly, our study revealed that specific deletion of C/EBPα in tubular epithelial cells prevented ferroptosis in a mouse model of diabetic kidney disease, resulting in a reduction in macrophage infiltration and renal fibrosis. This groundbreaking discovery established a link between C/EBPα and ferroptosis, demonstrating that C/EBPα could attenuate ferroptosis in renal tubular epithelial cells by modulating ACSL4 expression. Mechanistically, an increase in C/EBPα levels during DKD upregulated ACSL4 expression, leading to the accumulation of PUFA-CoA. Given the increased presence of free oxygen radicals in the DKD environment, lipid peroxidation occurred in renal tubules, ultimately resulting in cell death [33].

To further examine the therapeutic potential of C/EBPα in DKD, we used Fucosterol, a C/EBPα inhibitor, to treat db/db mice. Fucosterol, a phytosterol abundant in brown seaweed, could regulate lipid generation by inhibiting C/EBPα [34, 35]. Recent studies have highlighted the significant anti-inflammatory effects of Fucosterol on various diseases, including neurological disorders, lung fibrosis, and COVID-19 [36–39]. Remarkably, our research demonstrated that Fucosterol substantially alleviated renal fibrosis and inflammation in db/db mice. Furthermore, we observed that Fucosterol treatment reduced ACSL4 expression, suppressed lipid peroxidation, and consequently protected against ferroptosis in db/db mice. This study identified a novel function of Fucosterol in DKD beyond its known anti-inflammatory properties in other organs.

However, our study has certain limitations that warrant consideration. Firstly, as a control for DKD patients, we chose renal sections from MCD patients. It is important to note that while the primary pathological changes in MCD are concentrated in podocytes, renal tubules may also exhibit minor lesions [40]. Despite this, due to the unavailability of healthy renal tissue, MCD was chosen as a relatively normal control in our study. Secondly, specific probes for tissue sections, such as Liperfluo for live cells, were not available. Prussian blue staining showed no significant differences between healthy and DKD mice. Consequently, we used indirect indicators such as 4-HNE, MDA, and GPX4 to gauge ferroptosis levels in the DKD mouse model. Thirdly, C/EBPα is widely expressed in organs. However, due to time and research funding constraints, we only investigated the effects of Fucosterol on the kidney. Additionally, Fucosterol is not a specific inhibitor of C/EBPα. Previous studies have shown that fucosterol affects several pathways, making it challenging to determine whether fucosterol exclusively protects db/db mice from renal injury solely by inhibiting C/EBPα. However, given the absence of a specific inhibitor, Fucosterol remains the best available treatment option.

In summary, our research revealed a novel role of C/EBPα in tubular renal injury in DKD by modulating ACSL4 expression through direct binding to its transcriptional regulatory site. Fucosterol could inhibit C/EBPα, indicating a new target for preventing renal failure in DKD. Importantly, our findings were rigorously validated in vitro and in vivo. These results establish a solid foundation for future research endeavors in this field.

## Materials and methods

### Ethics approval and consent to participate statement

Animal experimental protocols were approved by the Animal Care Committee of Ruijin Hospital, Shanghai Jiao Tong University School of Medicine (Shanghai, China) and all methods were carried out in accordance with relevant guidelines and regulations (No. AUP-20240731-01).

All the studies on human tissues were approved by the Ethics Committee of Ruijin Hospital, Shanghai Jiao Tong University School of Medicine (Shanghai, China) (2020)瑞北伦审第(012)-1. Patients were consented by an informed consent process that was reviewed by the Ethics Committee of Ruijin Hospital that the study was performed in accordance with the ethical standards as laid down in the 1964 Declaration of Helsinki. There are no images from human research participants.

### Animal Models

All the mice were housed in a specific pathogen-free room at a constant temperature of 22 ± 2 °C and a constant humidity of 50 ± 5% under a 12-h day and night cycle. STZ/high-fat-diet (HFD)-induced diabetic nephropathy was induced in mice as previously described [41]. Mice with a C57BL/6 background do not develop DKD lesions after diabetes induction by STZ. Therefore, HFD is commonly used to induce obesity and insulin resistance in this strain. Six-week-old male mice randomly divided into control and DKD groups. DKD group mice were fed an HFD, followed by uninephrectomy and partial insulin deficiency induced by STZ injection. The mice were maintained on the HFD for a total of 16 weeks.

Spontaneous type 2 diabetic db/db mice: Homozygous BKS *db/db* mice (B6. BKS (D)- Lepr^db^/J, stock no. 000697) were purchased from Syagen Biology. Proteinuria was observed between the age of 8 weeks as a marker of the successful establishment of the type 2 DKD model. For treatment, 8-week-old db/db mice were administered 20 mg/kg of Fucosterol continuously for 6 weeks before being sacrificed.

Proximal tubular-specific C/EBPα-knockout mice were generated as described previously [21]. *Cebpa*^*fl/fl*^ mice were crossed with pepck-Cre mice. (provided by John Cijiang He, Icahn School of Medicine at Mount Sinai, New York, USA.) To confirm the knockout of Cebpa, we used genotyping, Western blot, and immunofluorescence (Fig. S1A to D). Both *Cebpa*^*fl/fl*^ and pepck-Cre mice were generated on a C57BL/6 J background. Mice with two wild-type alleles and Cre expression (*Pepck-Cre*^*+*^*/Cebpa*^*+/+*^) were used as controls.

### Cell culture and treatments

Mouse primary tubular epithelial cells (PTECs) were obtained as previously described [42]. All cells were synchronized to quiescence by being cultured in serum-free medium for 24 to 48 h and then treated with different stimuli as follows: (1) advanced glycation end products (AGEs) (0, 25, 50, or 100 μg/mL in culture medium), with medium containing an equal concentration of BSA as a control; (2) ICCB280 at concentration of 0.5 to 50 μmol/L in culture medium), with medium containing equal concentration of DMSO as a control.

### RNA sequencing

RNA sequencing was performed as previously described [43]. After treated with 50 μmol/L ICCB280 or DMSO, PTECs were harvested, and total RNA was extracted. Libraries were constructed using the VAHTS Universal V6 RNA-seq Library Prep Kit according to the manufacturer’s instructions. Transcriptome sequencing and analysis were carried out by OE Biotech Co., Ltd. (Shanghai, China). The libraries were sequenced on an Illumina NovaSeq 6000 platform, generating 150 bp paired-end reads. Clean reads were aligned using HISAT2. Differential expression analysis was conducted using DESeq2 (v 3.2.1). R (v 3.2.0) was used to construct column diagrams, chord diagrams, and bubble diagrams for the significantly enriched terms.

### Cell viability assay

Cell viability was assessed using the Cell Counting Kit-8 (CK04; Dojindo, Kumamoto, Japan) according to the manufacturer’s protocol. Approximately 0.8 × 10^4^ cells per well were plated in a 96-well plate. After treatment, 10 μL of CCK-8 solution was added to each well, and the cells were incubated for 3 hours. Cell viability was measured by determining the optical density at 450 nm (Beckman).

### Overexpression of C/EBPα

PTECs were transfected with either an empty vector (pcDNA3.1_3xFLAG) or a C/EBPα overexpression vector (pcDNA3.1_3xFLAG_*Cebpa*) for 24 hours using Lipofectamine 3000. After transfection, the cells were treated with D-glucose or AGEs.

### Western blot analysis

Protein samples were obtained from cells or tissue samples lysed using RIPA buffer (Millipore). The following antibodies were used: C/EBPα (Cell Signaling Technology, #8178S, 1:1,000), beta-actin (Cell Signaling Technology, #3700S, 1:2000), alpha-tubulin (Proteintech, #66031-1-Ig, 1:2,000), GAPDH (Proteintech, #60004-1-Ig, 1:2,000), FACL4 [EPR8640] (Abcam, #ab155282, 1:1,000), and glutathione peroxidase 4 [EPNCIR144] (Abcam, #ab125066, 1:1,000). Secondary antibodies used were from Cell Signaling Technology (anti-rabbit IgG, HRP-linked antibody, #7074, 1:5000; anti-mouse IgG, HRP-linked antibody, #7076, 1:5000). Relative protein levels were normalized to β-actin or α-tubulin according to the experimental conditions outlined in the figure legends using ImageJ.

### RNA extraction and real-time PCR

RNA extraction was performed using the Eastep Super Total RNA Isolation Kit (LS1040, Promega), and cDNA synthesis was carried out with the cDNA Synthesis Kit (R323, Vazyme) using approximately 400 ng of RNA. Quantitative PCR (qPCR) was conducted with ChamQ Universal SYBR qPCR Master Mix (Q711, Vazyme) on an ABI 7500 Real-Time PCR system and an ABI QuantStudio 6 (Applied Biosystems, Foster City, CA, USA) according to the manufacturer’s protocols. The 2^Δ–CT^ method was used to determine the relative mRNA levels.

### Glutathione measurements

Cellular glutathione levels were assessed using a GSH/GSSG kit (Beyotime, S0053) following the manufacturer’s instructions. Results were normalized to protein concentration, determined by the BCA assay.

### MDA assay

To quantify tissue MDA levels, a Lipid Peroxidation (MDA) Assay Kit (ab118970, Abcam) was used according to the manufacturer’s instructions. Values were normalized to tissue weight.

### Immunofluorescence staining

Immunofluorescence staining was performed as previously described [44]. Cells were incubated with primary antibodies, followed by secondary antibodies, and mounted. The primary antibodies used were anti-Aquaporin1 (Abcam, #ab9566, 1:200), anti-FACL4 (Abcam, #ab155282, 1:200), anti-C/EBPΑ (Cell Signaling Technology, #8178S, 1:100), and anti-4 Hydroxynonenal (Abcam, #ab46575, 1:200). Secondary antibodies were conjugated to FITC or PE (1:500). Images were obtained using standard or confocal microscopy, with counting and quantification were performed using the Cell Profiler program.

### Flow cytometry

PTECs were seeded in a 12-well plate at a density of 8.0 × 10^5^ cells per well. The cells were then treated with D-glucose, AGEs, or ICCB280 and subsequently detached using trypsin. For lipid peroxidation analysis, cells were incubated in 5 μM C^11^-BODIPY (D3861; Thermo Fisher) or 1 μM Liperfluo (L248; Dojindo) working buffer for 30 min at 37 °C in 5% CO_2_. Following incubation, the cells were washed twice with PBS and analyzed using CytoFlex S flow cytometer (Beckman). Data were processed and analyzed using FlowJo (BD Biosciences, Bedford, MA).

### Chip-qPCR

Chip-qPCR was performed as previously described [45]. Immunoprecipitation was conducted using an anti-C/EBPα antibody (8178S; Cell Signaling Technology) or rabbit IgG, followed by DNA purification and quantitative PCR analysis. The Enzymatic Chromatin IP Kit (9003, Cell Signaling Technology) was used according to the manufacturer’s instructions. Primers targeting two conserved C/EBPα-binding sites in the *ACSL4* promoter were used for amplification. The primer sequences were as follows:

478 base pairs upstream of the transcriptional start site: forward 5′-acaaagctgcggtgactttt-3′ and reverse 5′-tgcgttaagatccccgctc-3′;

1.456 kilobases upstream of the transcriptional start site: forward 5′-catcttcatccagcacactgat-3′ and reverse 5′-gcactatcacctacagttgggtt-3′.

### Statistics

There is no ramdomization or blinding in this study. Experimental data were analyzed using parametric tests unless otherwise specified. Differences between two independent groups were compared using a t-test. If unequal standard deviations (SDs) were present, Welch’s correction was applied. For comparisons involving more than two groups, ordinary one-way ANOVA was used. In cases of unequal SDs, the Brown-Forsythe and Welch ANOVA tests were used. A p-value less than 0.05 indicated statistical significance. Data analysis was conducted using GraphPad 8.4.0, and results are presented as means ± SDs.

#### Study approval

Animal maintenance and experimental procedures were approved by the Animal Care Committee of Ruijin Hospital, Shanghai Jiao Tong University School of Medicine (Shanghai, China). Approval for the use of human renal biopsy samples in this study was granted by the Ruijin Hospital Ethics Committee.

## Supplementary information

SUPPLEMENTAL MATERIAL

Uncut Blot Figures

## Data Availability

The microarray datasets have been deposited in the Gene Expression Omnibus under the accession code GSE242804. Other data that supporting the findings of this study are available from the corresponding author upon reasonable request.
