# Supplementary material for: C/EBPα-mediated ACSL4-dependent ferroptosis exacerbates tubular injury in diabetic kidney disease
Source: Cell Death Discov. 2024 Oct 23;10:448. doi: 10.1038/s41420-024-02179-w (PMC11499655; doi:10.1038/s41420-024-02179-w)
Supplement: Supplementary file 1 — SUPPLEMENTAL MATERIAL [file 41420_2024_2179_MOESM1_ESM.docx]

**Supplementary Figure S1: Confirmation of *Cebpa* deletion in mice.** (A) Immunoblotting and statistical analysis of C/EBPα protein level in the renal cortex of WT and Cebpa-KO mice. (B) Representative gel electrophoresis result of genotyping in mice. (C) Immunofluorescence and statistical analysis of C/EBPα protein level in the renal cortex of WT and Cebpa-KO mice. The data are presented as the mean ± SD; * P < 0.05, ** P < 0.01, and *** P <0.001 compared with the control.

**Supplementary Figure S2: C/EBPα expression was increased in glucose and AGEs treated PTECs.** (A) RT‒qPCR analysis of *Cebpa* mRNA levels in PTECs stimulated with 30 mM glucose for 48 h. (n= 4 per group) (B) Immunoblot and statistical analysis of C/EBPα protein levels in PTECs stimulated with 30 mM glucose for 48 h. (C) Immunoblot and statistical analysis revealed increased C/EBPα protein levels in PTECs stimulated with different concentrations of AGEs. (n = 3 per group). The data are presented as the mean ± SD; * *P* < 0.05, ** *P* < 0.01, and *** *P* <0.001 compared with the control.

**Supplementary Figure S3: Basement data of C/EBPα tubular-specific knockout mice.** (A and B) Mouse body weight (A) and blood glucose (B) levels. (n = 5-7 mice per group). (C) The kidney weight-to-body weight ratios of DKD Cre+/*Cebpa*^+/+^ and Cre+/*Cebpa*^f/f^ mice. (n = 5-6 mice per group). (D) RT‒qPCR analysis of *Havcr1* mRNA levels in DKD WT and *Cebpa*-KO mice. (n = 5-7 per group). (E) RT‒qPCR analysis of *Gpx4* mRNA levels in the renal cortex of DKD WT and *Cebpa*-KO mice. The data are presented as the mean ± SD. ns: no significant difference, ** *P* < 0.01, **** *P* < 0.0001 compared with the respective experimental control; ###*# P* < 0.0001 compared with cells under the same experimental conditions

**Supplementary Figure S4: Knockout and overexpression of Cebpa in mice kidneys.** (A) KEGG enrichment analysis of genes that were upregulated after ICCB280 stimulation. (B) Immunoblot and bar plot showing ACSL4 protein levels in kidneys from WT and *Cebpa*-KO mice. (n = 3 per group). (C) Representative IF staining of the kidneys of mice injected with PBS or AAV9-GFP-Vector. Scale bar: 20 μm. (D) The mRNA level of *Lcn2* in the kidney tissues of DKD control and *Cebpa*-overexpressing mice. (n = 5 mice per group). (E) The mRNA levels of *Col1a1, Col3a1,* and *Fn1* in kidney tissues from DKD control and *Cebpa*-overexpressing mice. (n = 5 mice per group). Bar graphs represent the mean ± SD. The data are presented as the mean ± SD. ns: no significant difference, * *P* < 0.05, ** *P* < 0.01, *** *P* <0.001 compared with the respective experimental controls.

**Supplementary Figure S5. Induction of C/EBPα exacerbated ferroptosis** **in PTECs.** (A) Representative layout of the Liperfluo fluorescence intensity of PTECs treated with ICCB280. (B) GSH levels in PTECs treated with 50 μM ICCB280 for 24 h.(n = 3 per group) (C) Representative MitoSOX staining of PTECs treated with 50 μM ICCB280 for 24 h. Scale bar: 30 μm. (D) the MitoSOX fluorescence intensity of PTECs treated with 50 μM ICCB280 for 24 h. (n = 3 per group). The data are presented as the mean ± SD. ns: no significant difference, * *P* < 0.05, ** *P* < 0.01, *** *P* <0.001 compared with the respective experimental controls.

**Supplementary Figure S6. Overexpression of *Cebpa* exacerbated ferroptosis in PTECs by upregulating ACSL4.** (A) The mRNA levels of *Acsl4, Por*, and *Lpcat3* in PTECs transfected with the pcDNA3.1 vector and pcDNA3.1-*Cebpa*. (B) Immunoblot and statistical analysis of ACSL4 protein levels in PTECs transfected with the pcDNA3.1 vector or pcDNA3.1-*Cebpa*. (C to E) Liperfluo fluorescence (C nad D), and GSH levels (E) in PTECs treated with D-glucose or AGEs after being transfected with the pcDNA3.1 vector or pcDNA3.1-*Cebpa*. (n = 3-5 per group) The data are presented as the mean ± SD. ns: no significant difference, * *P* < 0.05, ** *P* < 0.01, *** *P* <0.001 compared with the respective experimental controls; # *P* < 0.05, ### *P* < 0.0001 compared with cells under the same experimental conditions.
