# Supplementary material for: C/EBPα-mediated ACSL4-dependent ferroptosis exacerbates tubular injury in diabetic kidney disease
Source: Cell Death Discov. 2024 Oct 23;10:448. doi: 10.1038/s41420-024-02179-w (PMC11499655; doi:10.1038/s41420-024-02179-w)
Supplement: Supplementary file 2 — Uncut Blot Figures [file 41420_2024_2179_MOESM2_ESM.pptx]

## Slide 1
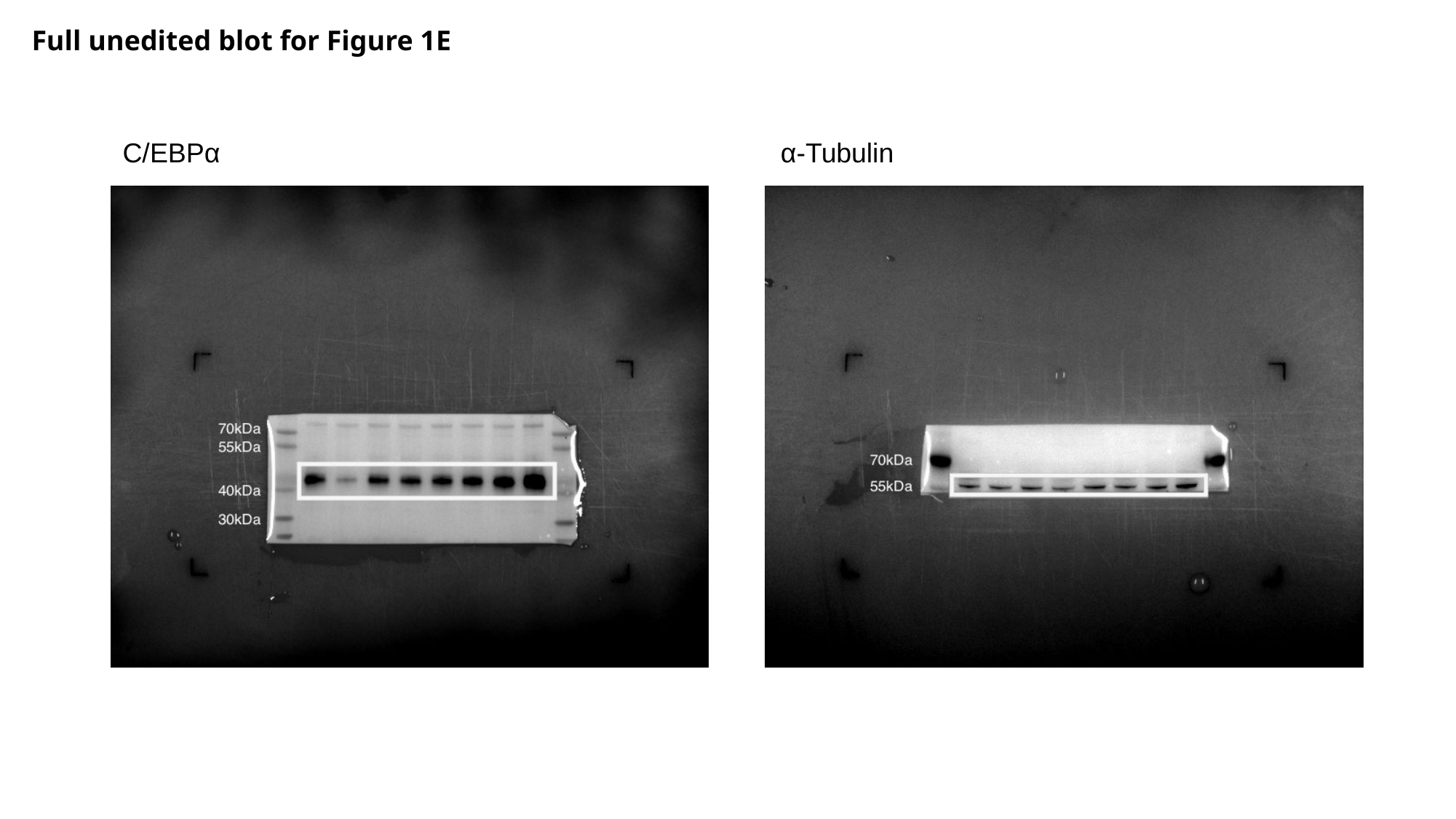

Full unedited blot for Figure 1E
C/EBPα
α-Tubulin

## Slide 2
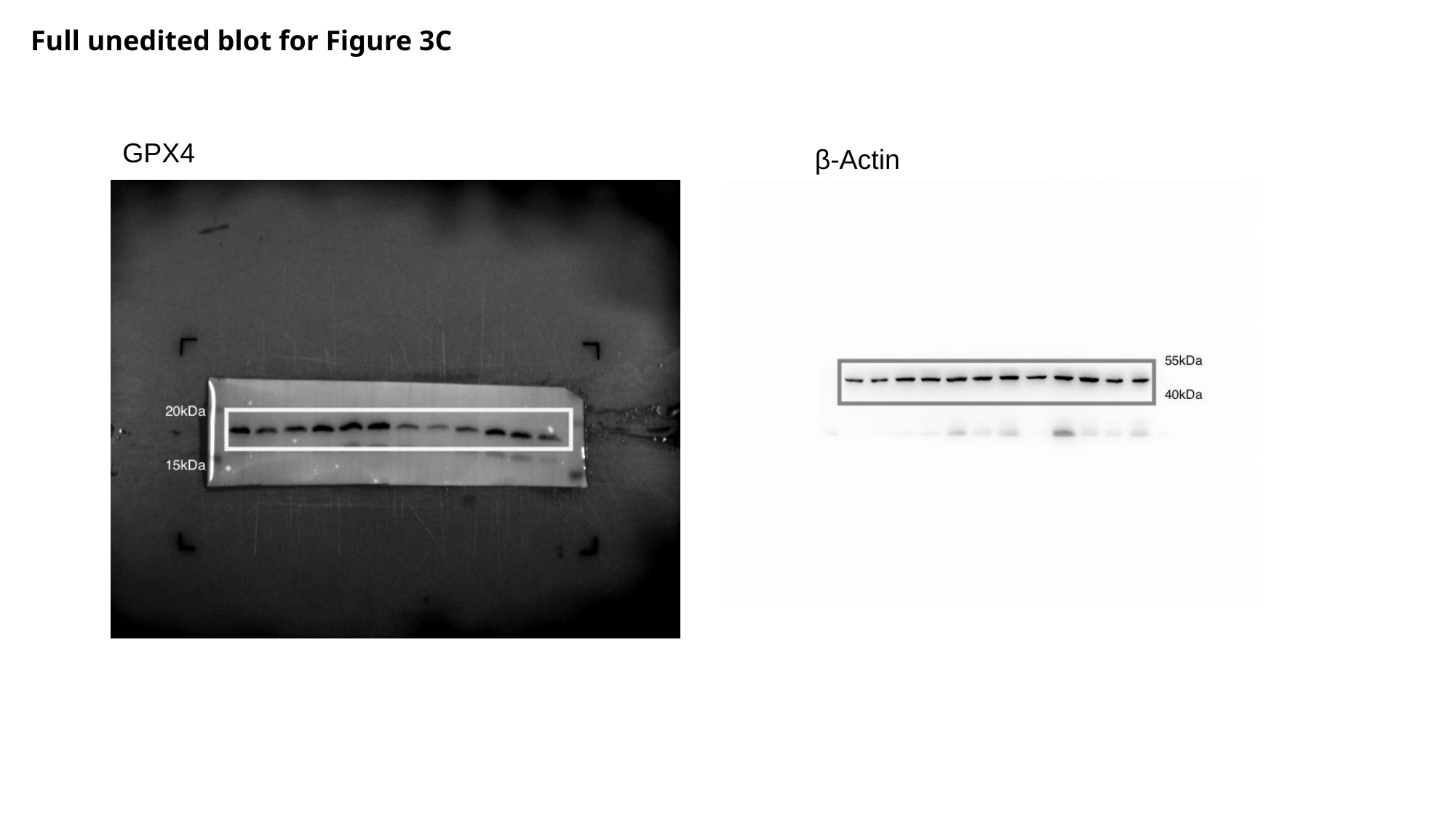

Full unedited blot for Figure 3C
GPX4
β-Actin

## Slide 3
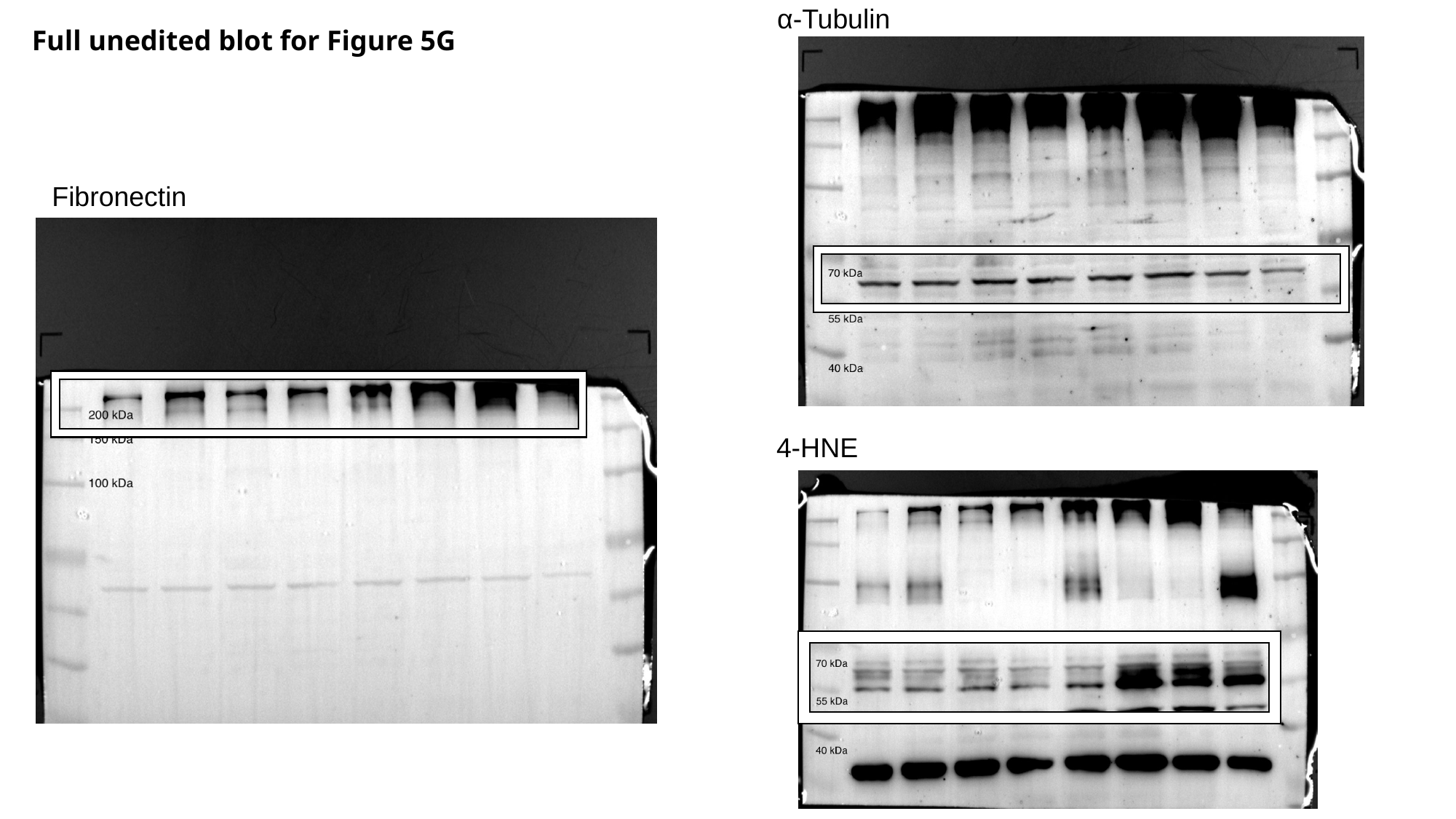

α-Tubulin
Full unedited blot for Figure 5G
Fibronectin
4-HNE

## Slide 4
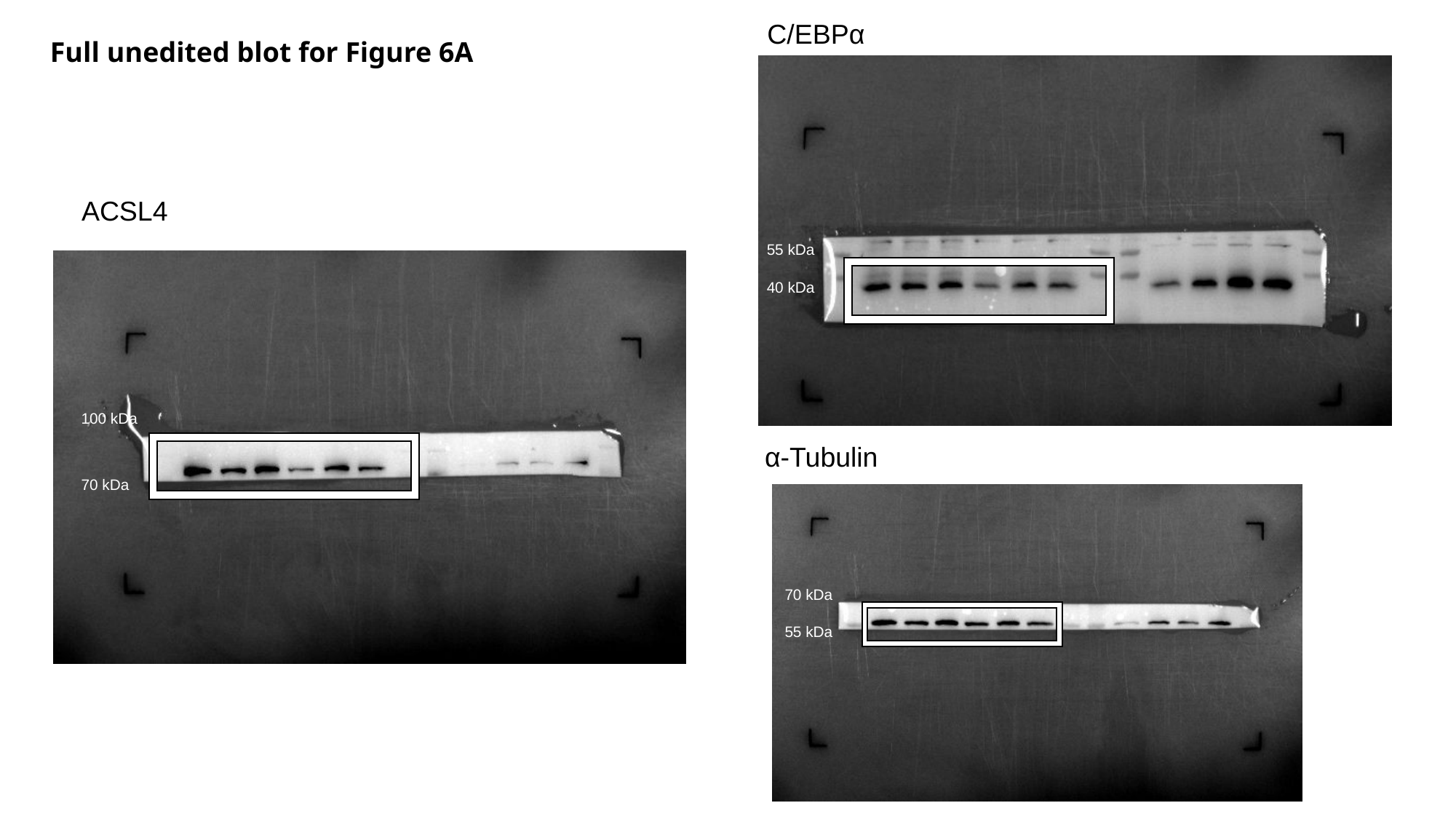

C/EBPα
Full unedited blot for Figure 6A
55 kDa
40 kDa
ACSL4
70 kDa
100 kDa
α-Tubulin
70 kDa
55 kDa

## Slide 5
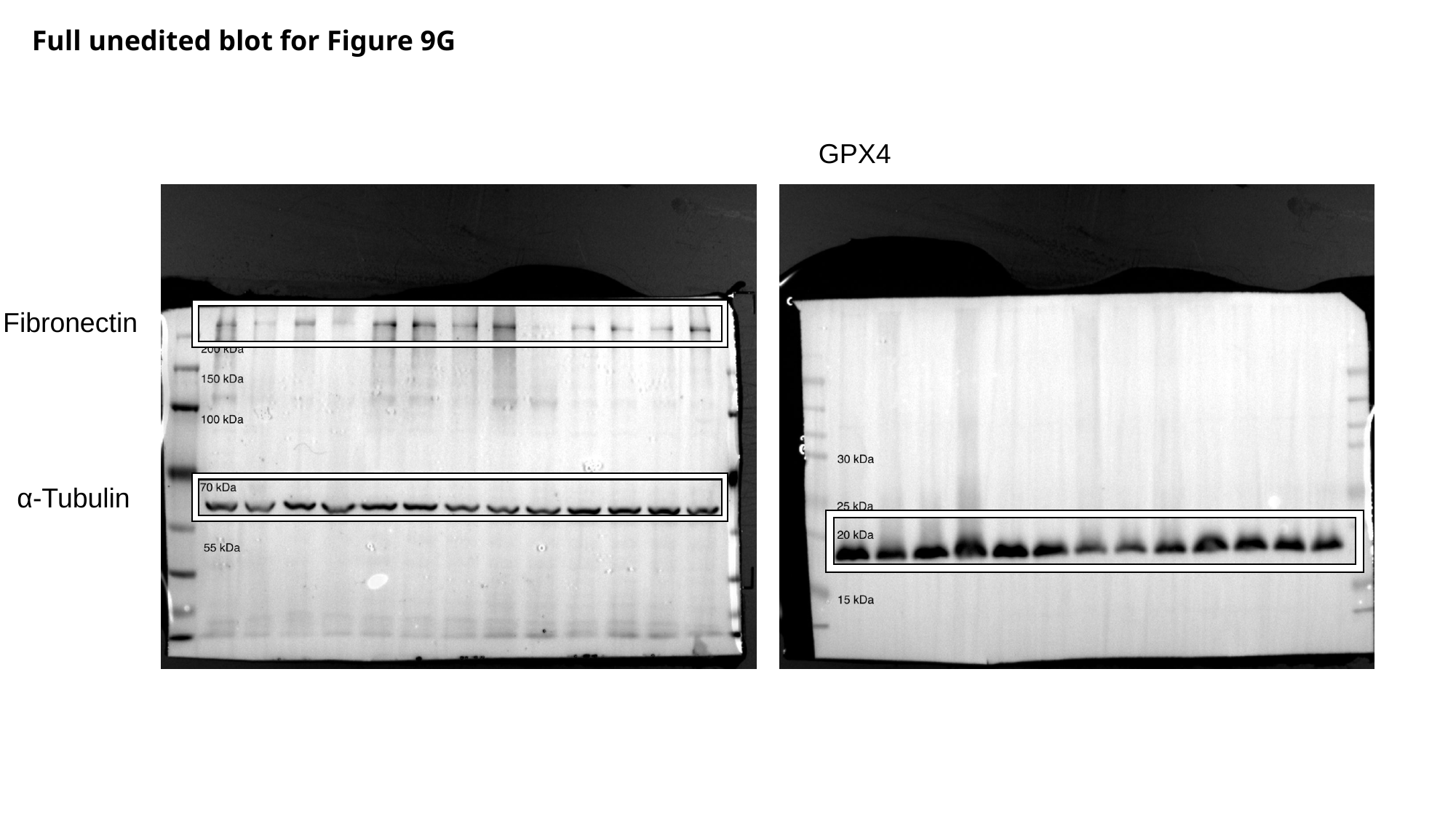

Full unedited blot for Figure 9G
GPX4
Fibronectin
α-Tubulin
